# Fatty acids, health lipid indices, and cholesterol content of sheep meat of three breeds from Moroccan pastures

**DOI:** 10.5194/aab-63-471-2020

**Published:** 2020-12-16

**Authors:** Kamal Belhaj, Farid Mansouri, Abdessamad Benmoumen, Marianne Sindic, Marie-Laure Fauconnier, Mohamed Boukharta, C. Hana Serghini, Ahmed Elamrani

**Affiliations:** 1 Laboratory for Improving Agricultural Productions, Biotechnology and Environment (LAPABE), Faculty of Sciences, University Mohammed First, 717, Oujda, 60000, Morocco; 2 Analysis Quality and Risk Unit, Laboratory of Food Quality and Safety (QSPA), Gembloux Agro-Bio Tech, University of Liège, Gembloux, 5030, Belgium; 3 General and Organic Chemistry Unit, Gembloux Agro Bio-Tech, University of Liège, Gembloux, 5030, Belgium; 4 Institute of Agricultural Industries, High School of Charlemagne, Huy, 4500, Belgium

## Abstract

The lipid analysis of commercial lamb's meat, from two main Moroccan production areas Middle Atlas and highlands of eastern Morocco, was conducted. This study concerns the lipid quality of sheep meat from Beni Guil (BG) and Timahdite (Ti) as indigenous breeds and that of the Ouled Djellal (ODj) breed of Algerian origin. To study the effect of the geographical area, the meat samples from the Beni Guil breed were taken in the two main locations of this breed: in the region of Ain Beni Mathar (BGA) and that of Tendrara (BGT). The fatty acid profiles of the sheep meats analyzed showed the following: (i) polyunsaturated fatty acid (PUFA) richness was of 12.87 % and 20.59 % respectively for BGA and BGT breeds; (ii) polyunsaturated / saturated fatty acid ratios were ranged between 0.28 to 0.50 respectively for BGA and Ti breed; and (iii) PUFA-n-3 content was 1.71 % for ODj breed and 2.13 % for BGA. Thus, the PUFA-n-6 / PUFA n-3 ratios range between 4.92 and 9.6 for BGA and Ti sheep meat, respectively. The cholesterol content was 0.08 % and 0.12 % of fresh meat respectively for ODj and BGA. Finally, meats of BGA and ODj have similar thrombogenicity (1.23 and 1.27 respectively) and atherogenicity indices (0.71 and 0.68 respectively). Statistically, these values were significantly higher than those registered for Ti breed (IT: 1.04 and AI: 0.51). In conclusion, from a nutritional point of view, it can be deduced that these meats have an interesting lipid quality due to their richness in desirable fatty acid (UFA + C18 : 0).

## 1 Introduction

In human nutrition, red meats often have a bad reputation among dieticians who consider them too rich in fat (Orellana et al., 2009). Cardiovascular diseases (CVDs) and cancer are among the most important causes of human mortality and are strongly related to a diet of cholesterol and saturated fatty acids (Wood et al., 2003). However, the intramuscular fat of meat is a calorically dense macronutrient which plays a key role in human nutrition due to its richness in essential fatty acids (Cabrera and Saadoun, 2014). Several studies showed that red meat from pasture has lower intramuscular fat and balanced ratios of lipid quality than animals from feedlots (Pordomingo et al., 2012; Saadoun et al., 2013). Indeed, recent studies showed that certain fatty acids (FAs) in these meats can have a very favorable effect on human health, such as rumenic acid (Howes et al., 2015). Human nutritionists, public health institutes, and various authorities are recommending balanced ratios between saturated fatty acids (SFAs) and unsaturated fatty acids (UFAs), especially of polyunsaturated FAs (PUFAs) n-3 in favor of n-6 FAs (Wood et al., 2004; Simopoulos and Dinicolantonio, 2016). Research around the world is aimed at assessing the meat quality through the evaluation of the proportions of essential FAs and other human health promoting FA, such as PUFA n-3 (Mortimer et al., 2014; Ponnampalam et al., 2014). Furthermore, the ratio of PUFA / SFA, PUFA n-6 / PUFA n-3 PUFA, hypo/hypercholesterolemic fatty acids, thrombogenicity index, and atherogenic index are important parameters to assess the nutritional quality of meat fat regarding the prevention of CVDs and cancer (Bressan et al., 2011; Troy et al., 2016).

In Morocco, 95 % of the sheep population is composed of local breeds (Beni Guil, Timahdite, Sardi, Boujaâd, and D'man), and its production is mainly based on pastures (Boulanouar and Paquay, 2006; Elbeltagy, 2012). The trend towards the lean meat consumption from pastures places the sheep meats of indigenous breeds in a favorable position compared to those of selected or imported breeds for a better production (breeds that have high productivity). This study concerns the evaluation of lipid quality of sheep meat from Beni Guil (BG) and Timahdite (Ti) as indigenous breeds and that of the Ouled Djellal (ODj) breed of Algerian origin, currently competing with BG in eastern Morocco highlands due to its weight characteristics.

## 2 Materials methods

### 2.1 Animal material

Sheep livestock in Morocco is generally destined for meat production where the majority of the males are intended for the feast of the sacrifice, and about 25 % of female lambs are reserved to replace the elderly ewes. The rest are fattened and intended for slaughter to supply the red meat markets throughout the year. In this study, 48 carcasses of female lambs from three sheep breeds were reared in Moroccan pastures. Two indigenous breeds (Beni Guil (n=24) and Timahdite (n=12)) and one non-indigenous breed of Algerian origin called Ouled Djellal (n=12), which cohabits with the Beni Guil sheep in the highlands of eastern Morocco, were used in this research.

Animals were aged between 7 and 8 months, weaned at the age of 3 months, of the L category according to the regulation of the European Commission no. 823/98. The animals weight at slaughter was between 33–37 kg, with an average-high fattening state, according to the EUROP community scale of grading sheep carcasses (Sagot and Pottier, 2011b) and the conformation ranged between fair (O) to good (R) class according to the EUROP grid (Sagot and Pottier, 2011a).

### 2.2 Breeding condition

The sheep farmers are members of the national association of breed producers (ANOC). They adopt a rhythm of lambing per year. Sampling was done with the assistance of the slaughterhouse veterinarian. Forty-eight longissimus lumborum muscles (LLM) were obtained 24 h after slaughter. These BG and ODj lambs belong to herds raised in the highlands of eastern Morocco, and the Ti lambs belong to herds raised in the rural commune of El Hajeb (Middle Atlas). The breeds used in this study were reared in the same production and rearing system. The animals in eastern Morocco are reared on natural highlands in an arid to a semi-arid environment. The diet of the ewes was composed of natural pastures when available and the same supplementation of 150 to 250 g per day based on alfalfa hay and barley in drought, hunger season, and in some physiological stages, such as at the time of preparation for breeding (flushing) and for lambing (steaming). The lambing season occurs mainly from September to December. Four weeks before parturition, ewes receive barley supplementation to avoid abortions, as well as to improve milk production and quality after lambing. The lambs are vaccinated 15 d after lambing for enterotoxemia. In the first two months of age, the lambs stay in the sheepfold during the day, giving them a supplement based on barley and alfalfa hay ad libitum. Then, in the third month of age, the lambs were raised with their mothers until weaning at the age of 3 months and grazed on natural grassland pastures with additional feeding based on barley (100–120 $g\;d^{-1}$) and hay. Before slaughter, the lambs undergo a finishing phase of 45 d based on barley (1 to 1.5 $\mathrm{kg}\;d^{-1}$). The lambs have free access to water and mineral supplement in the form of a lick block.

In the pastoral system adopted by breeders of eastern Morocco and the Middle Atlas, the adopted feeding calendar is dominated by grazing that lasts up to 8–12 $\mathrm{months}\;{\mathrm{yr}}^{-1}$ depending on climatic conditions (rainfall).

In the eastern region of Morocco, animals move south in winter and north in summer in the same area to graze on the halfah grass (*Stipa tenacissima*) and wormwood (*Artemisia herba-alba*) steppes. In July–August, the animals feed on cereal stubbles. The dominant plant species in the steppe and highland pasture of eastern Morocco were wormwood (*Artemisia herba-alba*), halfah grass (*Stipa tenacissima*), esparto grass (*Lygeum spartum*), *Atriplex halimus*, ray grass (*Lolium perenne* L.), laser white (*Laserpitium latifolium*), and sweet broom (*Arthrophytum scoparium*), with the presence of other species, such as *Bromus* spp. *Eruca vesicaria* (roquette), *Stipa capensis*, and *Medicago* spp. (Bechchari et al., 2014).

To study the effect of the geographical area, the meat samples from the Beni Guil breed were taken in the two main locations of this breed: in the region of Ain Beni Mathar (BGA) n=12 (-2.0247${}^{\circ }$ longitude, 34.0081${}^{\circ }$ latitude and 921 m altitude) and in that of Tendrara (BGT) n=12 (longitude: -2.0${}^{\circ }$, latitude: 33.04${}^{\circ }$ and altitude 1442 m).

In the Middle Atlas, the pair rangelands–forest is used throughout the year. At an altitude of 1500 m, breeders use stubble and crop residues and co-products from market gardening in summer, fallow in spring, and raw barley in winter. This is the case of the El Hajeb region (longitude -5.49${}^{\circ }$, latitude 33.69${}^{\circ }$, and altitude 773 m), where the samples of the Timahdite breed were taken. The dominant plant species in this geographical area were *Quercus rotundifolia* and *Q. ilex*, *Stipa tenacissima*, *Dactylis glomerata*, *Festuca* spp, *Cynosurus elegans*, *Crataegus laciniata*, *Arrhenatherum elatius*, and *Juniperus phoenicea*, with the presence of other species, such as *Avena* spp., *Papaver *spp., *Trifolium*, *Hordeum murinum*, and *Vicia* spp. (Berkat and Tazi, 2004).

### 2.3 Preparation of freeze-dried meat

The samples were frozen, lyophilized, crushed, and stored at -20 ${}^{\circ }C$ for subsequent analyses.

### 2.4 Dry matter and intramuscular fat (IMF) extraction

Dry matter was calculated using the drying method in a stove at 100±3 ${}^{\circ }C$ according to the method described by AOAC (1990). IMF was extracted and quantified according to Bligh and Dyer (1959) using a mixture of chloroform / methanol / water mixture (2/1/1; v/v/v).

### 2.5 Fatty acid analysis

The lipid extract was methylated before analysis. The FAs were converted to fatty acid methyl esters (FAMEs) according to the method described by Ben Moumen et al. (2015) using BF3 at 14 % weight in methanol. The separation of the FAMEs was performed on an Agilent gas chromatograph (GC) (HP6890 series, Agilent Technologies, USA) equipped with an Omega wax capillary column (30m×0.25mm, 0.25 m film thickness) from Supelco (Bellefonte, PA, USA) and a flame ionization detector (FID). Helium (99.999 %, Air Liquide, Liege, Belgium) was used as the carrier gas, at a flow rate of 1.7 $\mathrm{mL}\;\min^{-1}$. The temperatures of the injector and detector were set at 150 and 250 ${}^{\circ }C$, respectively, and the oven temperature was 210 ${}^{\circ }C$. The injection volume was 1 µL, in split-less mode. A FAME standard, containing 37 components (Supelco, Bellefonte, PA, USA), was used to identify the individual peaks. The average amount of each fatty acid was used to calculate the sum, ratios, and lipid indices of the meat.

### 2.6 Cholesterol content

The cholesterol analysis was carried out according to the method described by Vanderplanck et al. (2011). One gram of fat from each sample is saponified in the presence of 50 mL of methanolic KOH (2 N) at 80 ${}^{\circ }C$ for 1 h under reflux. After cooling, 1 mL of betulin solution was added as an internal standard (1 $\mathrm{mg}\;{\mathrm{mL}}^{-1}$). Then, the solution was diluted with 25 mL of distilled water. The extraction of the unsaponifiable fraction was carried out three times with 50 mL diethyl ether. Once the operation is completed, the ethereal extracts containing the unsaponifiable compounds were washed three times with 50 mL of distilled water and then dried over anhydrous sodium sulfate (${\mathrm{NaSO}}_{4}$). The diethyl ether was evaporated under reduced pressure at 35 ${}^{\circ }C$ until complete dry. After addition of 1 mL of chloroform, the fraction was separated by elution with a mixture of chloroform, diethyl ether, and ammonia water (90:10:0.5; V/V). The plates were allowed to dry and then were sprayed by 2.7-dichlorofluorescein 0.2 % ethanolic solution, followed by UV examination (254 nm). The bands corresponding to the sterols were recovered, and the sterols were re-extracted three times with 5, 3, and 3 mL of chloroform respectively. The obtained extracts were filtered, and the solvent was removed by evaporation with nitrogen. Next, the cholesterol was converted to trimethylsilyl ethers by the addition of 100 µL of a 1:1 (V/V) mixture of anhydrous pyridine and a silylation reagent (trifluoroacetamide and trimethylchlorosilane (BSTFA + TMCS) 99:1 (V/V) (Supelco, Bellefonte, PA, USA)), and then the mixture was left for 30 min at 90 ${}^{\circ }C$. The samples were evaporated with nitrogen and then taken up in 1 mL of hexane. Finally, the mixture was analyzed using gas chromatography (HP 6890 series GC) equipped with a capillary column HP 5 MS (5 %-phenyl-methylpolysiloxane, 30 m×0.25mm, 0.25 m film thickness) from Supelco (Bellefonte, PA, USA) and a flame ionization detector (FID).

### 2.7 Calculations

Saturated fatty acids (SFAs) were the sum of C14 : 0, C15 : 0, C16 : 0, C17 : 0, C18 : 0, C20 : 0, C22 : 0, C23 : 0, and C24 : 0. Monounsaturated fatty acids (MUFAs) were the sum of C14 : 1, C15 : 1, C16 : 1, C17 : 1, *cis*/*trans*-C18 : 1, C20 : 1n-9, and C22 : 1n-9. Polyunsaturated fatty acids (PUFAs) were the sum of n-3 (C18 : 3n3, C20 : 3n3, C20 : 5n3 and C22:6n3), n-6 (*cis*/*trans*-C18:2n6, C18:3n6, C20:3n6 and C20:4n6) fatty acids, C20 : 2, and C22 : 2. Unsaturated fatty acids (UFA) were the sum of MUFAs and PUFAs. Desirable fatty acids (DFAs) were C18 : 0 and UFA. Odd fatty acids (OFAs) were the sum of C15 : 0, C17 : 0, C17 : 1, and C23 : 0. Thrombogenic index (TI) = [C14 : 0 + C16 : 0 + C18 : 0]/[(0.5⋅MUFA) + (0.5⋅n-6) + (3⋅n-3) + (n-3/n-6)]. Atherogenic index (AI) = [(4⋅14 : 0) + 16 : 0]/[(AGPI) + (AGMI)] (Gama et al., 2013). Hypocholesterolemic fatty acids (h) were the sum of C18:1, C18:2n6, C20:4n6, C18:3n3, C20 : 5n3, C22 : 5n3, and C22 : 6ω3. Hypercholesteremic fatty acids (H) were the sum of C14 : 0 and C16 : 0. The h/H was calculated according to Fernández et al. (2007).

### 2.8 Statistical analysis

Experimental results were expressed as means ± SD of triplicate determinations. All statistical analyses were conducted using the Statistical Package for the Social Sciences (IBM SPSS 20). The normal distribution was verified according to the Shapiro–Wilk test. One-way ANOVA statistical analysis and Tukey's post hoc test were used for means comparison; the difference was considered significant at p<0.05. Principal component analysis (PCA) was performed on the chemical data set in order to verify whether it was possible to differentiate the samples according to their meat quality characteristics and to obtain more information on the variables that mainly influence the fatty acid and cholesterol content.

**Table 1:** Moisture, intramuscular fat (IMF), and cholesterol content of Beni Guil (BG), Timahdite (Ti), and Ouled Djellal (ODj) sheep meats from Moroccan pastures.

Parameters	Breed				Effect
(% fresh matter basis)	BGA	BGT	Ti	ODj	
Moisture	73.17±1.34	73.85±0.94	73.41±1.27	73.63±0.76	NS
IMF	$5.73\pm 1.31^{b}$	$4.80\pm 1.00^{a}$	$5.5\pm 1.30^{b}$	$4.60\pm 0.88^{a}$	NS
Cholesterol	$0.12\pm 0.02^{b}$	$0.11\pm 0.01^{b}$	$0.10\pm 0.01^{\text{a, b}}$	$0.08\pm 0.01^{a}$	**

Values in the same row with different superscripts are significantly (p<0.05) different. NS: not significant; ${}^{*}$ p<0.05; ${}^{**}$ p<0.01; ${}^{***}$ p<0.001; BGA: female lambs of Beni Guil breed sampled in the Ain Beni Mathar region; BGT: female lambs of Beni Guil breed sampled in the Tendrera region.

## 3 Results and discussion

### 3.1 Fatty acid profile and cholesterol content

An increasingly important aspect of meat quality is its nutritive value, especially fatty acid composition (Milićević et al., 2014). As a matter of fact, according to recent research, individual or group FAs, namely SFAs, MUFAs, and PUFAs, are present in meat; their ratios and sums are emerging as a key factor in nutrition and health (Vannice and Rasmussen, 2014). Human nutritionists, public health institutes, and various authorities recommend reducing the consumption of SFAs and cholesterol (Yousefi et al., 2012). Red meat lipids are widely criticized for their saturated fatty acid richness (Geay et al., 2002). However, some fatty acids in beef and lamb have health benefits, such as *trans* vaccenic acid and long-chain PUFA n-3 (Howes et al., 2015).

Table 1 does not show a significant difference between the three studied breeds concerning the IMF content (4.6 per 5.73 %); meanwhile, the cholesterol content is significantly high in the BG breed regardless of its pasture (0.12 %–0.11 %) compared to Ti (0.1 %) and ODj (0.08 %) breeds. The obtained results are in line with the results of several authors (Martínez-Cerezo et al., 2005; Blasco et al., 2019) reporting no variation in intramuscular fat content according to genotype. In accordance with the above results, Vacca et al. (2008) and Sinanoglou et al. (2013) have reported a significant effect of genotype on cholesterol content. However, no effect of breed on the cholesterol content was observed but rather an effect of the diet. Valsta et al. (2005) reported that the cholesterol concentration of meats range between 30 and 120 mg/100 g of meat portion. Our results are lower than those found in the meat of the crossbreed mouflon × Sarda (356 mg/100 g of fresh matter); however, they are higher than those reported by Yousefi et al. (2012) for two Iranian breeds (50.08–53.37 mg per 100 g of meat) and by Liu et al. (2015) for Oula lamb meat (54.84–60.24 mg per 100 g of meat). Similar to these findings, Sinanoglou et al. (2013) reported a cholesterol concentration of 90.71 mg/100 g FM in the meat of Karagouniko lamb. According to Kalogeropoulos et al. (2004), the European Olive Oil Medical Information and the World Health Organization have set a maximum daily cholesterol dose of less than 300 mg per day. Thus, the contribution of cholesterol, by consumption of 100 g of examined sheep meat, represented only 28 % to 39 % of the recommended maximum cholesterol intake.

The gas chromatography analysis of the FA composition allowed the separation and the identification of 26 fatty acids (Table 2). The main LLM fatty acids of the analyzed meats are, in descending order, oleic acid (32.06 %–37.62 %), palmitic acid (20.72 %–25.02 %), stearic acid (12.38 %–13.78 %), and linoleic acid (7.37 %–11.4 %). Saturated fatty acids are often considered undesirable for our health (Wood et al., 2008). However, much research has reported the beneficial effect of the same SFA, such as stearic acid and short-chain FAs (Dietschy, 1998; Bonanome and Grundy, 1988). Stearic acid, in comparison, is assumed to have no effect on blood cholesterol levels and is considered as a good predictor of cover fat firmness of carcasses (Wood et al., 2004). Overall, our results show the prevailing individual saturated FAs were palmitic acid (PA), stearic acid (SA), and myristic acid (MA). The highest values of SFAs were recorded in BGA (46.53 %) and ODj (42.98 %) sheep meat. This difference is mainly related to the proportion of palmitic acid and myristic acid (PA: 25.02 %, 22.96 %, 20.72 % and 24.23 %; MA: 4.62 %, 4.34 %, 2.39 % and 3.53 % respectively for BGA, BGT, Ti, and ODj sheep meat). Ruminant meat is characterized by its high content of SFA due to the ruminal bio-hydrogenation phenomenon (Ben Abdelmalek et al., 2020). However, several studies showed that it is possible to vary the FA composition by varying the diet and the rearing system (Wood et al., 2003; van Harten et al., 2016; Margetín et al., 2018; Belhaj et al., 2020). The differences in SFA content are more due to the rearing system than the difference in genotype. Indeed, it can be deduced that the meats of BGA and ODj, which were raised in the same rearing system (semi-extensive of Ain Beni Mathar) with an important concentrate and halfah-based supplementation, are the richest in SFA. This high content of PA in BG and ODj meats is possibly due to the steppe–pasture diet rich in palmitic acid. In this context, Berrighi et al. (2017) had reported that the chemical composition of the steppe pasture diet contains 24.08 % to 28.48 % of PA. However, our results show no difference between the studied meats in the percentage of stearic acid. The obtained results of SFAs are lower than those reported by Margetín et al. (2018) in the meat of Île-de-France sheep breed and are comparable to those reported by Polidori et al. (2017) in the meat of the Fabrianese breed. The SFAs recorded values in LL muscle of Ti and BGT comparable to those found by Garcia et al. (2008) in the meat of Patagonian lamb.

**Table 2:** Fatty acid profiles of Beni Guil (BG), Timahdite (Ti), and Ouled Djellal (ODj) sheep meats from Moroccan pastures.

Fatty acid (%)	Breed				Effect
	BGA	BGT	Ti	ODj	
C14 : 0	$4.62\pm 1.41^{c}$	$4.34\pm 0.75^{\text{b, c}}$	$2.39\pm 0.69^{a}$	$3.53\pm 0.57^{b}$	***
C14 : 1	$0.37\pm 0.10^{a}$	$1.50\pm 0.19^{c}$	$0.32\pm 0.17^{a}$	$1.01\pm 0.40^{b}$	***
C15 : 0	0.58±0.11	0.58±0.10	0.51±0.07	0.53±0.20	NS
C15 : 1	$0.12\pm 0.05^{a}$	$1.27\pm 0.60^{c}$	$0.24\pm 0.03^{a}$	$0.80\pm 0.13^{b}$	***
C16 : 0	$25.02\pm 2^{c}$	$22.96\pm 0.83^{b}$	$20.72\pm 1.25^{a}$	$24.23\pm 2.02^{\text{b, c}}$	***
C16 : 1	$1.67\pm 0.29^{c}$	$0.90\pm 0.24^{b}$	$1.14\pm 0.17^{b}$	$0.79\pm 0.29^{a}$	***
C17 : 0	$1.24\pm 0.10^{b}$	$0.80\pm 0.13^{a}$	$1.74\pm 0.43^{c}$	$1.10\pm 0.16^{b}$	***
C17 : 1	$0.9\pm 0.05^{b}$	$0.14\pm 0.02^{a}$	$1.31\pm 0.28^{c}$	$0.15\pm 0.05^{a}$	***
C18 : 0	13.78±2.32	12.38±1.28	12.98±0.77	12.73±1.89	NS
*Cis*/*trans*-C18 : 1n9	$37.3\pm 2.78^{b}$	$32.06\pm 4.84^{a}$	$36.26\pm 1.77^{b}$	$37.62\pm 3.92^{b}$	***
*Cis*/*trans*-C18 : 2n6	$7.37\pm 2.11^{a}$	$10.68\pm 1.44^{b}$	$11.40\pm 1.56^{c}$	$7.90\pm 1.80^{a}$	***
C18 : 3n6 (ALA)	$0.83\pm 0.24^{a}$	$1.75\pm 0.34^{b}$	$0.68\pm 0.30^{a}$	$0.78\pm 0.07^{a}$	***
C18 : 3n3 (LA)	$0.90\pm 0.2^{b}$	$0.99\pm 0.30^{b}$	$0.49\pm 0.19^{a}$	$0.63\pm 0.18^{a}$	***
C20 : 0	$0.23\pm 0.03^{\text{b, c}}$	$0.1\pm 0.03^{a}$	$0.16\pm 0.05^{\text{a, b}}$	$0.29\pm 0.05^{c}$	***
C20 : 1n9	$0.13\pm 0.03^{a}$	$0.4\pm 0.10^{c}$	$0.17\pm 0.03^{a}$	$0.31\pm 0.07^{b}$	***
C20 : 2	$0.26\pm 0.10^{a}$	$1.47\pm 0.48^{c}$	$0.35\pm 0.08^{a}$	$0.60\pm 0.20^{b}$	***
C20 : 3n6	$0.19\pm 0.07^{a}$	ND	$0.36\pm 0.07^{b}$	ND	***
C20 : 3n3	$0.08\pm 0.0.02^{a}$	$0.14\pm 0.04^{b}$	$0.09\pm 0.03^{a}$	$0.19\pm 0.06^{c}$	***
C20 : 4n6 (ARA)	$2.09\pm 0.98^{a}$	$4.59\pm 0.63^{b}$	$5.52\pm 1.37^{b}$	$4.62\pm 0.63^{b}$	***
C20 : 5n3 (EPA)	$0.39\pm 0.20^{a}$	ND	$0.55\pm 0.20^{b}$	ND	*
C22 : 0	0.19±0.04	ND	0.17±0.03	ND	NS
C22 : 1n9	0.11±0.05	ND	0.11±0.06	ND	NS
C22 : 2	ND	0.25±0.09	ND	0.36±0.14	NS
C23 : 0	$0.17\pm 0.06^{a}$	$1.67\pm 0.48^{b}$	$0.48\pm 0.22^{c}$	$0.84\pm 0.17^{d}$	NS
C24 : 0	$0.62\pm 0.29^{b}$	$0.29\pm 0.14^{a}$	$1.09\pm 0.30^{c}$	$0.16\pm 0.06^{a}$	***
C22 : 6n3 (DHA)	0.76±0.13	0.72±0.16	0.74±0.26	0.89±0.22	NS

Values in the same row with different superscripts are significantly (p<0.05) different. NS: not significant; ND: not detected; ${}^{*}$ p<0.05; ${}^{**}$ p<0.01; ${}^{***}$ p<0.001; BGA: female lambs of Beni Guil breed sampled in the Ain Beni Mathar region; BGT: female lambs of Beni Guil breed sampled in the Tendrera region.

The MUFA content (Table 3) does not show a significant difference between BGA, Ti, and ODj; on the other hand, a significant lower content was observed for BGT. This difference is notably due to the C18 : 1 content (dominant MUFA), which was considered to be beneficial FAs for decreasing plasma cholesterol and low-density lipoprotein (LDL) (Tejeda et al., 2008; Reddy et al., 2015). Furthermore, our estimates of enzymatic activity of Delta9-desaturase C18 responsible for conversion of C18 : 0 to their cis-9 monounsaturated, obtained by product-substrate ratios, did not differ significantly among the studied meats (data not shown). Thus, in the case of BGT, it is assumed that the difference in C18 : 1 content would not be due to an insufficient synthesis of C18 : 1 from C18 : 0 by desaturation but rather to a less important provision in C18 : 0, to a greater desaturation of C18 : 1 to C18 : 2, or to a more significant bio-hydrogenation of C18 : 1 to C18 : 0. However, the latter hypothesis should also be ruled out since no difference in the C18 : 0 level was observed for the BGT meats compared to the other studied meats. Significant differences (p<0.05) were recorded in the other minor MUFAs such as myristoleic acid (C14 : 1), 10-pentadecenoic acid (C15 : 1), palmitoleic acid (C16 : 1), and 11-eicosenoic acid (C20 : 1n-9).

**Table 3:** Partial sums of fatty acids (g per 100 g fatty acids), ratios, and indices of Beni Guil (BG), Timahdite (Ti), and Ouled Djellal (ODj) sheep meats from Moroccan pastures.

Parameters	Breed				Effect
	BGA	BGT	Ti	ODj	
SFA	$46.45\pm 1.70^{c}$	$43.12\pm 2.15^{b}$	$40.24\pm 1.86^{a}$	$43.41\pm 4.05^{b}$	***
MUFA	$40.6\pm 5.04^{b}$	$36.27\pm 3.52^{a}$	$39.55\pm 2.86^{b}$	$40.68\pm 3.57^{b}$	***
PUFA	$12.87\pm 3.61^{a}$	$20.59\pm 3.61^{c}$	$20.18\pm 2.21^{c}$	$15.61\pm 2.71^{b}$	**
UFA	$53.47\pm 1.7^{a}$	$56.86\pm 1.66^{b}$	$59.73\pm 1.68^{c}$	$56.29\pm 4.05^{b}$	***
DFA	$67.25\pm 3.20^{a}$	$69.24\pm 0.37^{a}$	$72.71\pm 2.5^{b}$	$69.02\pm 2.59^{a}$	***
OFA	$2.99\pm 0.13^{a}$	$4.46\pm 0.67^{b}$	$4.27\pm 0.76^{b}$	$3.42\pm 0.44^{a}$	***
UFA/SFA	$1.15\pm 0.07^{a}$	$1.32\pm 0.08^{b}$	$1.48\pm 0.11^{c}$	$1.30\pm 0.19^{b}$	***
PUFA/SFA	$0.28\pm 0.08^{a}$	$0.48\pm 0.1^{b}$	$0.50\pm 0.15^{c}$	$0.36\pm 0.08^{\text{a, b}}$	***
PUFA n-6	$10.48\pm 3.31^{a}$	$17.02\pm 1.29^{c}$	$17.96\pm 1.29^{c}$	$13.3\pm 2.89^{b}$	***
PUFA n-3	$2.13\pm 1.28^{b}$	$1.86\pm 0.46^{\text{a, b}}$	$1.87\pm 0.41^{\text{a, b}}$	$1.71\pm 0.31^{a}$	*
n-6/n-3	$4.92\pm 1.74^{a}$	$9.16\pm 2.7^{b}$	$9.60\pm 3.18^{b}$	$7.77\pm 2.7^{b}$	***
TI	$1.36\pm 0.10^{c}$	$1.23\pm 0.11^{b}$	$1.04\pm 0.08^{a}$	$1.27\pm 0.17^{\text{b, c}}$	***
AI	$0.83\pm 0.15^{c}$	$0.71\pm 0.04^{\text{c, b}}$	$0.51\pm 0.08^{a}$	$0.68\pm 0.12^{b}$	***
h/H	$1.73\pm 0.26^{a}$	$1.94\pm 0.10^{b}$	$2.47\pm 0.25^{c}$	$1.96\pm 0.28^{b}$	***
Nutritive value of fat	$2.05\pm 0.22^{a}$	$1.93\pm 0.11^{a}$	$2.39\pm 0.21^{b}$	$2.09\pm 0.21^{a}$	***

Values in the same row with different superscripts are significantly (p<0.05) different; BGA: female lambs of Beni Guil breed sampled in the Ain Beni Mathar region; BGT: female lambs of Beni Guil breed sampled in the Tendrera region. SFA: saturated fatty acids; UFA: unsaturated fatty acids; PUFA: polyunsaturated fatty acids; DFA: desirable fatty acids (C18 : 0 + UFA); OFA: odd fatty acids; IT: thrombogenic index = [C14 : 0 + C16 : 0 + C18 : 0]/[(0.5⋅MUFA) + (0.5⋅∑n-6) + (3⋅∑n-3) + (n-3/n-6)]. IA: atherogenic index = [(4⋅C14 : 0) + C16 : 0]/[(PUFA) + (MUFA)], calculated as per Ulbrichtet Southgate (1991) without the inclusion of C18 : 0, which is considered to be neutral on serum cholesterol; hypocholesterolemic FA (h)/hypercholesterolemic FA (H) = (18 : 1n9c + 18 : 2ω6 + 20 : 4ω6 + 18 : 3ω3 + 20:5ω3 + C18:3ω6 + C20 : 3ω6 + 20 : 2 + 22 : 6ω3)/(14:0 + 16 : 0); Nutritive value of fat = (C18 : 0 + C18 : 1)/C16 : 0.

Sheep meat is among the sources of essential fatty acids, especially linolenic acid (ALA, n-3) and long-chain PUFA n-3, including eicosapentaenoic acid (EPA, C20 : 5 n-3) and docosahexaenoic acid (DHA, C22 : 6 n-3), which have potential benefits concerning heart health (Howes et al., 2015). In this study, the predominant PUFAs in analyzed meats are C18 : 2n-6 (7.37 %–11.40 %) and C20 : 4n6 (2.09 %–5.52 %). Belhaj et al. (2020) reported similar results for BG sheep meat produced in Ain, Beni Mathar region. The proportion of the PUFAs was significantly higher in the muscle of BGT (20.2 %) and Ti (20.61 %) lambs than in BGA (12.87 %) and ODj (15.89 %) lambs. Regarding the sums of PUFAs n-3, the results show a significant difference between the studied meats, where the higher value was recorded in BGA lambs (2.13 %) and the lower in ODj lambs (1.71 %). For BGA and ODj breeds, the obtained results of PUFAs are higher in comparison with those reported by Garcia et al. (2008) for Patagonian (13.85 %) lambs and are lower than those reported by Margetín et al. (2018) for Île-de-France (17.21 %) and by van Harten et al. (2016) for Dorper lambs (16.93 %). Nevertheless, the recorded PUFA values in BGT and Ti lambs are higher than those reported by Margetín et al. (2018) for Île-de-France (16.93 %) lambs and similar to the result obtained by van Harten et al. (2016) for Merino (21.85 %) lambs and by Addis et al. (2013) for Agnello di Sardegna (21.85 %) lambs.

**Figure 1:**
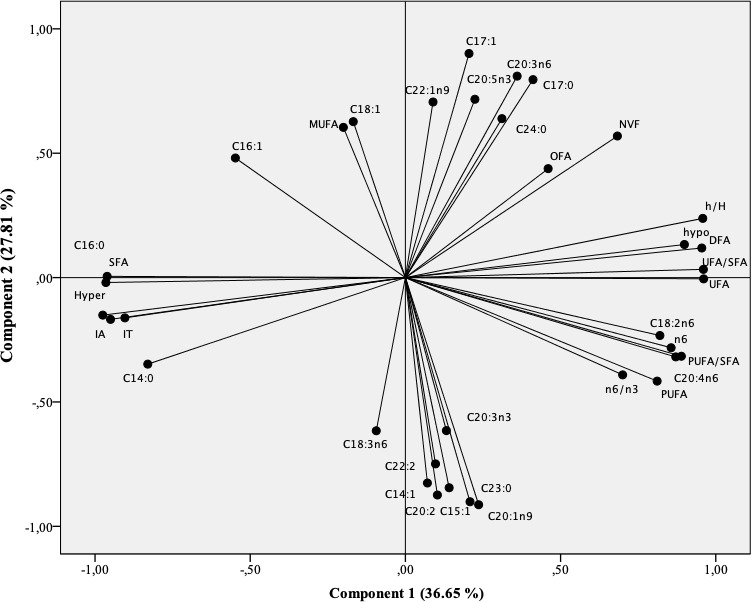
Projection of fatty acids and health lipid indices of intramuscular fat in the plane defined by two principal components.

The ratios and indices of FAs classes as affected by breed are presented in Table 3. According to Sinanoglou et al. (2013), these ratios and indices are important keys when assessing the nutritional value of meat fat in regard to the prevention of CVDs and carcinogenicity (Cabrera and Saadoun, 2014). The PUFA / SFA ratios range from 0.28 for BGA breed meat to 0.50 for Ti breed meat. These recorded ratios (0.28 vs. 0.36 vs. 0.48 vs. 0.50 for BG, ODj, BGT and Ti respectively) are around the recommended value in human nutrition (0.45) (Wood and Enser, 1997). Thus, the values found in this study were higher than those found by Garcia et al. (2008) for Patagonian lambs (0.35) in Argentina, by Maia et al. (2012) for meat of crossbred lambs (0.2–0.3) in Brazil, by Yousefi et al. (2012) for two Iranian breeds (0.16–0.19), by Liu et al. (2015) for Oula lambs in China, and by Berrighi et al. (2017) for meat reared in highland and steppe in Algeria. Similarly, Faria et al. (2012) reported PUFA / SFA ratios varied between 0.43 and 0.55, and Sinanoglou et al. (2013) reported values between 0.37 and 0.49, which are lower than those reported for Fabrianese lambs by Polidori et al. (2017). This difference can be attributed to different age at slaughter and to the fact that the studied animals were functional as ruminants, whereas Polidori et al. (2017) used young animals, which are not yet functional as ruminants.

**Figure 2:**
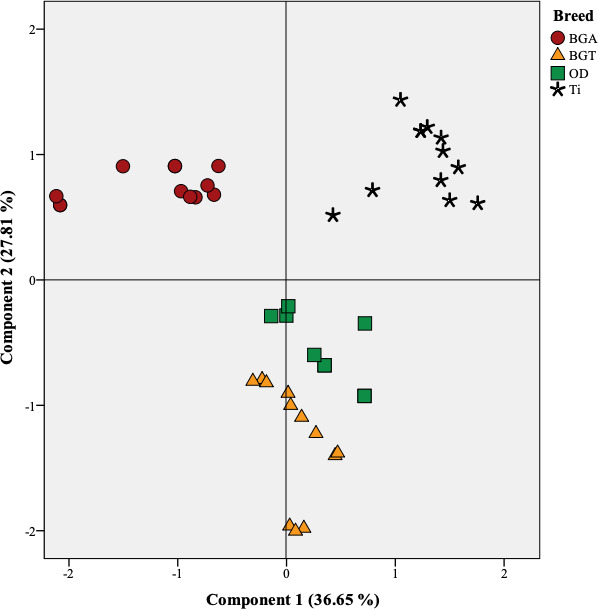
Projection of the variables of the four studied meats in the plane defined by two principal components; BGA: female lambs of Beni Guil sheep breed sampled in the Ain Beni Mathar region; BGT: female lambs of Beni Guil sheep breed sampled in the Tendrera region; ODj: Ouled Djellal sheep breed; Ti: Timahdite sheep breed.

Therefore, Timahdite and BGT meats show the highest PUFA/SFA ratio and are richer in PUFA, which are not only beneficial for health but also for the development of oxidation products during cooking, which will contribute to the great meat flavor. The analysis of the PUFA / SFA ratio results also shows the influence of farming type (way) and feeding on the lipid profile. Thus, the meat of BG breed has a higher PUFA / SFA ratio when the farming method is more extensive in the Tendrera region (BGT; 0.48), where wormwood (*Artemisia herba-alba*) and halfah (*Stipa tenacissima*) are not degraded compared to Ain Beni Mathar breeding area (BGA; 0.28), where they are in an advanced degradation state (Bechchari et al., 2014). As a result, breeders in Ain Beni Mathar adopt a semi-extensive rearing method with the use of barley and alfalfa hay supplementation. The richness of the food ration in wormwood (antimicrobial and antioxidant) and halfah, which is rich in unsaturated fatty acids, would be responsible for this difference (Mehdadi et al., 2006; Pérez-Alvarez and Fernández-López, 2009). *Artemisia* is rich in active compounds such as antimicrobials that inhibit certain ruminal bacteria, and therefore they would reduce stomach bio-hydrogenation, especially tannins. Thus, they would reduce the oxidative degradation of PUFAs due to the richness of their essential oils in antioxidants (Pérez-Alvarez and Fernández-López, 2009; Vasta et al., 2013; Benouda et al., 2018). The ODj sheep meat has a similar ratio (PUFA / SFA: 0.36) to that of the BGA breed, since it was raised practically with the same rearing system: semi-extensive of Ain Beni Mathar region. The meat of Ti breed would have the highest PUFA / SFA and n-6 / n-3 ratio, because of the forest–pasture nature, which is characterized by a diet that includes green oak (*Quercus Ilex*) rich in PUFA. Various studies have already shown the beneficial effect of a diet rich in oak acorns on the meat quality from different domestic animal species (Keddam et al., 2010; Ramdane, 2014; Mekki et al., 2019). Indeed, the incorporation of acorns rich in antioxidants especially in tocopherol makes it possible both to improve the PUFA content and to reduce their oxidative degradation, therefore preserving their nutritional quality (Akcan et al., 2017). In addition, this decrease in oxidation would improve the meat color (Jerónimo et al., 2016; Mekki et al., 2019). The presence of tannins in acorns would improve the PUFA / SFA ratio by reducing ruminal bio-hydrogenation. The obtained results correspond to those obtained by several other authors who reported an increase in the PUFA / SFA ratio in free grazing (Wood et al., 2003; Margetín et al., 2018). Thus, the finishing phase of barley-based lambs influences the content of PUFAs. However, the reported results of Sauvant and Bas (2001) and Popova et al. (2015) showed that a concentrated carbohydrate-rich diet would shorten the time food stays in the rumen (congestion time), thereby decreasing their bio-hydrogenation and therefore a reduction in SFA.

The analysis of other calculated lipid indices from the different obtained fatty acids values and which would have a nutritional effect shows significant differences between the four studied meats. From a nutritional point of view, the ratio n-6 / n-3 would be among the most important criteria of fat quality. Indeed, according to Simopoulos (2002) this ratio (n-6 / n-3) plays an essential role regarding the risk of atherosclerosis, especially the formation of blood clots leading to a heart attack (Simopoulos, 2002; Howes et al., 2015). The calculation of this ratio shows significant differences between the analyzed meats. Thus, the BGA meat has the lowest ratio (4.92), which is close to the limit values recommended by nutritionists (<4) (Sinanoglou et al., 2013; Howes et al., 2015). The ODj breed meat reared under the same conditions of BGA has a higher ratio of n-6 / n-3 (7.77) compared to the nutritional recommendations but lower compared to those obtained for Ti and BGT meats (9.5 and 9.15 respectively). The low value of the n-6 / n-3 ratio in ODj and especially BGA meats can be partly explained by the fact that the received food supplement is especially halfah rich in C18:3n3 fatty acid. Our study carried out in 2016 on the BG breed meat from the Ain Beni Mathar region had already shown the interest of this meat from a nutritional point of view (Belhaj et al., 2018). In fact, the values of the PUFA n-6 / PUFA n-3 ratio found in the four studied meats were considerably high (ranging from 4.92 in the BGA lambs to 9.6 in the meat of the Ti breed), considering the fact that the n-6 / n-3 ratio intake by humans should not exceed the value of 4 (Simopoulos, 2002; Howes et al., 2015). Similar values have been reported by Berrighi et al. (2017) in Reimbi lamb (crossbreed between BG breed and ODj breed) meats (8.4–12.68) and by Nasri et al. (2011) and Vasta et al. (2013) for meat of Barbarine breed (value around 7). However, the recorded values were lower than those reported by Blasco et al. (2019) for Segureña lambs (15.32–15.47) and were higher than those found by Yousefi et al. (2012) and Margetín et al. (2018) for Chall and Île-de-France sheep breed. Similar results were reported by Keddam et al. (2010) for n-6 / n-3 ratio of ODj breed reared in Algeria (8.97 for ODj with a barely diet and 7.37 for ODj with an oak acorn diet).

According to Sinanoglou et al. (2013), AI and TI should not exceed 1. In this study, the AI-obtained values are below the recommended value and are comparable to those reported by Sinanoglou et al. (2013) for Greek breeds (0.55–0.73) and by Costa et al. (2018) for lamb meat in Brazil. However, they are lower than those reported by Della Malva et al. (2016) for Altamurana lamb, by Oriani et al. (2005) for Merino (1.35) and by Margetín et al. (2018) for Île-de-France lambs (0.97). Timahdite meat has the lower AI ratio but the highest n-6 / n-3 ratio. This difference can be explained by the fact that AI takes into account both the content of some saturated fatty acids, which would have a harmful effect on human health, and contents of MUFA and PUFA with a beneficial effect in the prevention of vascular accidents. Concerning the TI, the obtained values are higher than the recommended value (1.04–1.36) (Sinanoglou et al., 2013). Palmitic and oleic acids represent the principal total fatty acids; the ratio (C18 : 0 + C18 : 1) / C16 : 0 is an important criterion to predict the meat lipid quality and is defined as the nutritional value of meat (Maia et al., 2012; Yaranoglu and Ozbeyaz, 2019). In this study, the obtained nutritional values range between 1.93 and 2.39 for BGT and Ti sheep breed respectively. These values are in accordance with those reported by Banskalieva et al. (2000). They reported that, in a normal case, the ratio (C18 : 0 + C18 : 1)/C16 : 0 found in the literature ranged between 2 and 3 for lamb meat. Thus, our results are comparable to those reported by Liu et al. (2015) and Yaranoglu and Ozbeyaz (2019) for Oula lambs (1.75–2.46) and for Turkish lambs (2.49–2.67), respectively. Regarding the sums of DFA, the results show a significant difference (p<0.01), with higher values recorded in the meat of Ti lambs (72.71 %). Our results are similar to those reported by Costa et al. (2018) for lambs in Brazil (72.3 %), to those reported by Yaranoglu and Ozbeyaz (2019) for Turkish lambs (70.64 %–72.10 %), and to the values reported by Banskalieva et al. (2000) for lamb meats (63.97 %–71.81 %). However, they are higher than those reported by Díaz et al. (2003) for Manchega lambs (42.3 %).

### 3.2 Principal component analysis

Principal component analysis (PCA) was conducted to provide an easy visualization of the relationships among fatty acid profile and health lipid indices of the studied meats. This analysis was used to further explore the above results. The results for PCA applied to parameter values are summarized in Figs. 1 and 2. Two principal components were extracted from statistical analysis, which explained 63.17 % of the total variance in the data set. The first and second PCs (PC1 and PC2) accounted for 36.65 % and 27.81 % of the variation, respectively. The PC1 was mainly characterized by hypocholesterolemic fatty acids, PUFA/SFA ratio, PUFA n-6, and C20 : 4n-6 on the right side, and SFA, AI, TI, and hypercholesterolemic fatty acids on the left side (Fig. 1). The PC2 was defined positively by MUFA especially n-9 (C18 : 1n9 and C22 : 1n9) and by C20 : 2, C22 : 2 and C23 : 0 in the opposite direction. The individual projections on the factorial map (Fig. 2) show discrimination between the studied meats allows summarizing the interpretations already mentioned above in a very simplified way. In fact, BGA lambs were located on the left side of the Fig. 2 and were clearly differentiated from all lamb breeds. Ti lambs were clearly differentiated from the other lamb types and are located on the right side of the Fig. 22, where PUFA, h/H, PUFA n-6, and P / S ratios lie. According to the PC2, the effect of geographical area is mainly manifested. In fact, the BGA was opposed to BGT on the second PC. The animals sampled in Ain Beni Mathar region are characterized by MUFA richness compared to those sampled in the Tendrera region.

PCA analysis exposed clearly the breed and geographical area effects on the fatty acid composition and intramuscular fat quality of the studied meats. Therefore, the breed and geographical area impact leads to significant differences in lipid quality (p<0.05), which most probably results from the feeding effect and genetic differences, as already mentioned by relevant studies in sheep and cattle (Zhang et al., 2008; Matsuhashi et al., 2011).

## 4 Conclusions

All obtained results clearly show that significant differences are noted on the lipid composition of the longissimus lumborum muscle of the studied breed in Moroccan livestock. The intramuscular fat content does not seem to be affected in this study either by race or by the farming method. Analysis of the fatty acid composition and lipid indices shows that meat of Ti breed has the best lipid profile with its richness in PUFA, its low value of AI, and its high DFA content. The richness in PUFA can be explained by its agrosilvopastoral rearing mode or/and by the pasture quality, rather than a difference in racial origin. Among the four studied breeds, the Ti breed is the only one whose diet is rich in oak acorns. Nevertheless, further studies are needed to confirm these results and to ascertain the mechanisms of action of Moroccan medicinal plants such as wormwood and halfah in eastern Morocco and acorn oak in the Middle Atlas on lipid metabolism in ruminants to determine the origin of this richness in PUFA.

## Data Availability

The original data of the paper are available upon request to the corresponding author.
